# Apolipoprotein J/Clusterin Is a Novel Structural Component of Human Erythrocytes and a Biomarker of Cellular Stress and Senescence

**DOI:** 10.1371/journal.pone.0026032

**Published:** 2011-10-06

**Authors:** Marianna H. Antonelou, Anastasios G. Kriebardis, Konstantinos E. Stamoulis, Ioannis P. Trougakos, Issidora S. Papassideri

**Affiliations:** 1 Department of Cell Biology and Biophysics, Faculty of Biology, University of Athens, Panepistimiopolis, Athens, Greece; 2 Department of Medical Laboratories, Faculty of Health and Caring Professions, Technological and Educational Institute of Athens, Athens, Greece; 3 Blood Transfusion Center, Nikea, Piraeus, Greece; Universidade de São Paulo, Brazil

## Abstract

**Background:**

Secretory Apolipoprotein J/Clusterin (sCLU) is a ubiquitously expressed chaperone that has been functionally implicated in several pathological conditions of increased oxidative injury, including aging. Nevertheless, the biological role of sCLU in red blood cells (RBCs) remained largely unknown. In the current study we identified sCLU as a component of human RBCs and we undertook a detailed analysis of its cellular topology. Moreover, we studied the erythrocytic membrane sCLU content during organismal aging, in conditions of increased organismal stress and accelerated RBCs senescence, as well as during physiological *in vivo* cellular senescence.

**Methodology/Principal Findings:**

By using a combination of molecular, biochemical and high resolution microscopical methods we found that sCLU is a novel structural component of RBCs extra- and intracellular plasma membrane and cytosol. We observed that the RBCs membrane-associated sCLU decreases during organismal aging or exposure to acute stress (e.g. smoking), in patients with congenital hemolytic anemia, as well as during RBCs *in vivo* senescence. In all cases, sCLU reduction paralleled the expression of typical cellular senescence, redox imbalance and erythrophagocytosis markers which are also indicative of the senescence- and oxidative stress-mediated RBCs membrane vesiculation.

**Conclusions/Significance:**

We propose that sCLU at the mature RBCs is not a silent remnant of the erythroid precursors, but an active component being functionally implicated in the signalling mechanisms of cellular senescence and oxidative stress-responses in both healthy and diseased organism. The reduced sCLU protein levels in the RBCs membrane following cell exposure to various endogenous or exogenous stressors closely correlates to the levels of cellular senescence and redox imbalance markers, suggesting the usefulness of sCLU as a sensitive biomarker of senescence and cellular stress.

## Introduction

Mammalian red blood cells (RBCs) have a unique structure, composition and functional properties that allow them to efficiently fulfil their crucial role in the maintenance of tissues homeostasis. Although mature RBCs represent a simplified cell type, they retain a number of molecular components of signalling and/or regulatory pathways [1]. Determination of the RBCs lifespan is a complex process affected by many cellular parameters. Specifically, the aging process of RBCs is characterized by cell shrinkage, membrane remodelling, micro-vesiculation and exposure of surface removal markers that trigger erythrophagocytosis [2]-[4]. Powerful removal signals are the externalization of phosphatidylserine and the binding of autologous immunoglobulins G (IgGs) to senescence-specific neo-antigens that originate from structural changes in the protein Band 3 [5]. The process of RBCs senescence is also associated with the operation of an apoptosis-like cell death program probably mediated by calpains and caspases activation [6]-[8]. As in the typical mammalian cells, a range of mechanisms that are responsive to oxidative stress seem to drive normal RBCs senescence *in vivo* [5], [9].

Secretory Apolipoprotein J/Clusterin (sCLU) is a heterodimeric disulfide-linked glycoprotein of ∼75-80 kDa being encoded by a single copy gene [10]. It functions at High Density Lipoprotein particles as an apolipoprotein [11]. Also, it has been shown that it binds to hydrophobic regions of partially unfolded proteins and via an ATP-independent mechanism inhibits protein aggregation and precipitation [12]. On the basis of this latter property, sCLU was classified as a functional homologue of the small Heat Shock Proteins (sHSPs) [13]. Considering this latter property it is not surprising that sCLU has being involved in several physiological processes including development [14], lipid transportation [11], differentiation [15], cellular senescence, *in vivo* aging as well as in many age-related diseases including neurodegeneration, vascular damage, diabetes and tumorigenesis [16]. As a result sCLU has attracted significant biomedical interest [10] being currently an antisense target in Phase III clinical trials in prostate cancer patients [17], while *CLU* gene variants were recently found to associate with Alzheimer's disease [18], [19]. Considering that the only common characteristic shared by all these, otherwise unrelated in their etiology and/or clinical manifestation, pathological conditions is the fact that they are all characterized by increased oxidative stress and injury, we recently proposed that sCLU is a sensitive cellular biosensor of oxidative stress that functions to protect cells from the deleterious effects of free radicals and their derivatives [16], [20].

The elucidation of signalling mechanisms operating during RBCs senescence or exposure to endogenous or exogenous stress are of great interest in cases of anemia, organism aging, exposure to noxious factors and blood banking. The appropriate intervention to those mechanisms could favourably affect both RBCs survival and functional competence. In view (1) of the functional implication of sCLU in cellular senescence and pathological conditions of increased oxidative injury, including organism aging [16] and (2) our previous preliminary studies showing sCLU localization in human RBCs [21] we investigated the probable role of sCLU in mature human healthy and stressed erythrocytes. In the present report we provide novel evidence showing that sCLU distributes in RBCs cytosol and membrane and that its relative content during senescence or in diseases closely correlates to the expression of typical cellular senescence, erythrophagocystosis and oxidative stress markers. Our novel findings clearly imply a functional role for sCLU in the physiology of human RBCs as a sensitive molecular biomarker of senescence and redox imbalance.

## Materials and Methods

### Ethics

The study has been submitted and has been approved by the Research Bioethics and BioSecure Committee of the Faculty of Biology/University of Athens. Investigations were carried out in accordance with the principles of the Declaration of Helsinki. Written informed consent was obtained from all blood donors participating in this study.

### Subjects

Venous blood of 45 healthy adult volunteers was used in the present study. In this cohort, 37 were non-smoking adults while 8 were heavy smokers. The non-smoking group consisted of young (N = 13, 20-28 years old, on average 24.1±2.2y), middle (N = 14, 30-45 years old, on average 39.1±4.3y) or old (N = 10, 74-87 years old, on average 82±4.9y) age subjects. The cigarette smokers were all of middle age (N = 8, 36-42 years old, on average 39.4±2.4y) and have been consuming two or more packets of cigarettes per day for 22.4±3.2 years. Blood samples from adult patients with hemolytic anemia (N = 10) who have been clinically diagnosed for mild or typical hereditary spherocytosis (HS, N = 5) [HS-1 (splenectomised), -4 and -5 with primary defects in spectrin; HS-2, -3 with primary defects in ankyrin], heterozygous 4.1(-) hereditary elliptocytosis (N = 2) and beta+ thalassemia/sickle cell anemia (N = 3) were collected in ethylenediaminetetraacetic acid (EDTA) and heparin. All patients were in good health and none presented transfusion-dependent anemia. Healthy non-smoking subjects were used as controls.

### Erythrocytes isolation and preparation of lysates, plasma membranes, cytoskeletons and vesicles

RBCs were isolated by the method of Beutler [22]. Briefly, leukocytes and platelets were removed from the blood samples by filtering through columns of α-cellulose and microcrystalline cellulose mixture (1∶1 by weight) in isotonic saline, phosphate buffered saline (PBS) or (4-(2-hydroxyethyl)-1-piperazineethanesulfonic acid (Hepes)-buffered isotonic saline (NaCl 133 mM, KCl 4.5 mM and Hepes 10 mM, pH 7.4) supplemented with protease inhibitors. RBCs were extensively washed and diluted to an appropriate hematocrit; in some cases a gentle negative pressure was applied to facilitate filtration. Purified RBCs were lysed with hypotonic (5 mM) sodium phosphate buffer (pH 8.0) containing a cocktail of protease inhibitors and membrane or cytosol fractions were prepared as previously described [23]. RBCs cytoskeletons were prepared from the washed membranes by Triton X-100 extraction [24]. Protein concentration was determined by using the Bradford protein assay.

### *In vitro* biotinylation, proteinase K treatment and oxidation of purified RBCs

For RBCs labelling with biotin, 2×10^8^ leukodepleted RBCs samples were surface labelled with 2 mM Sulfo-NHS-LC biotin in PBS, according to the manufacturer's instructions. For the proteinase K experiments, 2×10^8^ intact RBCs were digested with proteinase K (100 µg/ml) for 1 h at 37°C in PBS and were subsequently washed with ice-cold PBS containing protease inhibitors. RBCs intracellular proteinase digestions were carried out under identical conditions except that Nonidet P-40 (NP-40, 0.005% in PBS) was added in the digestion and washing buffers. RBCs were then pelleted, osmotically lysed and processed to biochemical fractionation into membrane and cytosol fractions (see above). For the *in vitro* oxidation experiments 30 ml of purified RBCs per sample were exposed to 2.5 mM t-butyl hydroperoxide (tBHP) in PBS. Following incubation for 30 min at 37°C the cells pelleted and the supernatant was centrifuged to collect the RBC-derived membrane vesicles.

### Isolation of young and senescent RBCs through fractionation according to cellular density

RBCs fractionation according to density was performed by means of a Percoll discontinuous gradient as described previously [25], [26]. Briefly, the gradient was built up in five layers of Percoll medium varied between 1.087 and 1.098 g/ml buffered with Hepes buffer solution pH 7.4, containing 5.25% (w/v) bovine serum albumin. The RBCs' suspension (40% hematocrit in Hepes-buffered isotonic saline) was layered on the top of the gradient and the fractions were collected by low speed centrifugation (2500 g) for 30 min at room temperature. Cells were excessively washed with Hepes buffer to remove Percoll and a range of age-dependent RBC parameters were recorded in each fraction (mean cellular volume MCV, mean cellular hemoglobin and mean cellular hemoglobin concentration MCHC) by means of an automatic blood cell counter (Sysmex K-4500, Roche).

### Immunoblotting analysis, detection of protein carbonyl groups and of Reactive Oxygen Species (ROS)

Equal amounts (12-200 µg) of RBCs fractions were loaded in Laemmli gels, blotted to nitrocellulose membranes and probed with primary and horseradish peroxidise (HRP)-conjugated secondary antibodies as previously described [27]. Immunoblots were developed using an enhanced chemiluminescence (ECL) reagent kit and quantified by scanning densitometry (Gel Analyzer v.1.0 image-processing program). Purified RBCs plasma membrane proteins were processed for the detection of carbonyl groups using the Oxyblot® detection kit as per manufacturer's specifications. For quantification purposes, the proteome carbonylation index (PCI) was calculated as described previously [28].

ROS accumulation in RBCs was detected with the membrane-permeable and redox-sensitive dye 5-(and-6)-chloromethyl-2′,7′-dichlorodihydrofluorescein diacetate, acetyl ester (CM-H_2_DCFDA) as per the manufacturer's guidelines with minor modifications [28]. More specifically, isolated and thoroughly washed RBCs (in triplicates) were incubated with pre-warmed PBS in the absence (endogenous ROS) or in the presence (exogenous oxidation) of 100 µM tBHP at 25°C. Following the removal of the oxidant (if applied), 1% RBCs suspension was loaded with 10 µM CM-H_2_DCFDA in PBS buffer for 30 min. Samples were then washed and incubated in the same buffer for 10-15 min in order to render the dye responsive to oxidation. Fluorescent dichlorofluorescein (DCF) was measured using the VersaFluor™ Fluorometer System (Bio-Rad: Excitation, 490 nm; Emission, 520 nm). The fluorescent intensity was normalized to the total protein level. The following negative controls were used: (1) unstained RBCs incubated with only PBS buffer to detect autofluorescence, and, (2) cell-free mixtures of dye and buffers with or without tBHP.

### Confocal Laser Scanning Microscope (CLSM) immunofluorescence and Transmission Electron microscopy (TEM) immunogold localization

Immunofluorescence assays were performed as previously described [8]. Briefly, RBCs were fixed with 90% methanol in PBS, permeabilized in PBS containing 0.05% Triton X-100 and blocked with 3% bovine serum albumin and 0.1% Tween-20 in PBS. Cells were probed with the appropriate primary and secondary antibodies conjugated to fluorescein isothiocyanate (FITC) or rhodamine. Slides were observed under a Digital Eclipse C1 (Nikon, Melville, NY) CLSM and recorded at the same exposure time. Controls were prepared as described previously [23] and showed no immunoreactivity. Shown micrographs are representative from RBCs derived from three different donors and six independent experiments.

For immunoelectron microscopy, RBCs were fixed in 4% paraformaldehyde, 0.1% glutaraldehyde in PBS pH 7.2 and embedded in unicryl acrylic resin as described previously [21], [23]. Thin sections were blocked with 3% fatty acid-free bovine serum albumin in PBS; probed with the sCLU antibody and immunoglobulins conjugated to 15 nm gold particles and examined on a Phillips EM 300 electron microscope operating at 80 kV accelerating voltage. Controls were as for CLSM and were all negative.

### Statistical analysis

Presented experiments have been repeated at least two times, unless otherwise stated. Data points correspond to the mean value; error bars denote standard deviation (s.d.). Individual protein levels were quantified against a reference RBCs membrane protein or against the sum of the normal proteins further normalized to the respective corresponding controls (healthy, young or non-smoking subjects and young or untreated cells). For statistical analysis the MS Excel and the Statistical Package for Social Sciences (IBM SPSS; version 19.0 for Windows; administrated by NKUA) were used. Significance was evaluated using the one-way analysis of variance (ANOVA). Comparisons between different groups were performed by the independent t-test or the chi-squared test. Spearman's correlation test (two-sided) was used to assess the relationship between variables (correlation coefficient r). Significance was accepted at p<0.05. P<0.05 or p<0.01 are indicated in the graphs by one or two asterisks, respectively.

### Material Supplies

The monoclonal antibodies against Band 3 (B9277) and actin (A5316), the polyclonal antibodies against spectrin (S1515) and human IgGs (A8792) and the HRP-conjugated secondary antibodies (A-5420), as well as the Protease Inhibitor Cocktail, tBHP, α-cellulose, microcrystalline cellulose (Sigmacell type 50), Percoll medium and common chemicals and buffers were obtained from Sigma-Aldrich (Germany). Polyclonal antibodies against hemoglobin (Hb) (GR800GAP) were obtained from Europa Bioproducts (Cambridge). Primary antibodies against CD47 (sc-25773), CLU (sc-6419), Hsp70 (sc-1060) and Band 3 (sc-20657) as well as secondary antibodies conjugated to fluorescein isothiocyanate or rhodamine were from Santa Cruz Biotechnology (Santa Cruz, CA). CM-H2DCFDA was from Invitrogen, Molecular Probes (C-6827), and streptavidin, HRP-conjugated secondary antibodies (NA 934) as well as enhanced chemiluminescence Western blot detection kit were from GE Healthcare Amersham (Piscataway, NJ). HRP-conjugated secondary antibodies (P0161) were from DakoCytomation (Glostrup, Denmark); Sulfo-NHS-LC biotin (21327) was from Pierce Biotechnology, Thermo Scientific (Rockford, USA) and Proteinase K was from Boehringer Mannheim (Germany). The Oxyblot® detection kit (S7150) was obtained from Millipore, Chemicon (Temecula, CA). Unicryl acrylic resin and IgGs conjugated to 15 nm gold particles were obtained from British Biocell International (Cardiff, Wales, UK). Bradford protein assay was from Bio-Rad (Hercules, CA). Gel Analyzer v.1.0 image-processing system and software was obtained from Biosure (Athens, Greece). The monoclonal antibody against stomatin and the antiserum against protein 4.1R were kindly provided by Prof. R. Prohaska (Department of Medical Biochemistry, Medical University of Vienna, Austria) and Prof. J. Delaunay (Service d' Hématologie, Hôpital de Bicetre, Le Kremlin-Bicetre, France) respectively.

## Results

### sCLU is a novel structural component of human RBCs plasma membrane and cytosol

**Figure 1:**
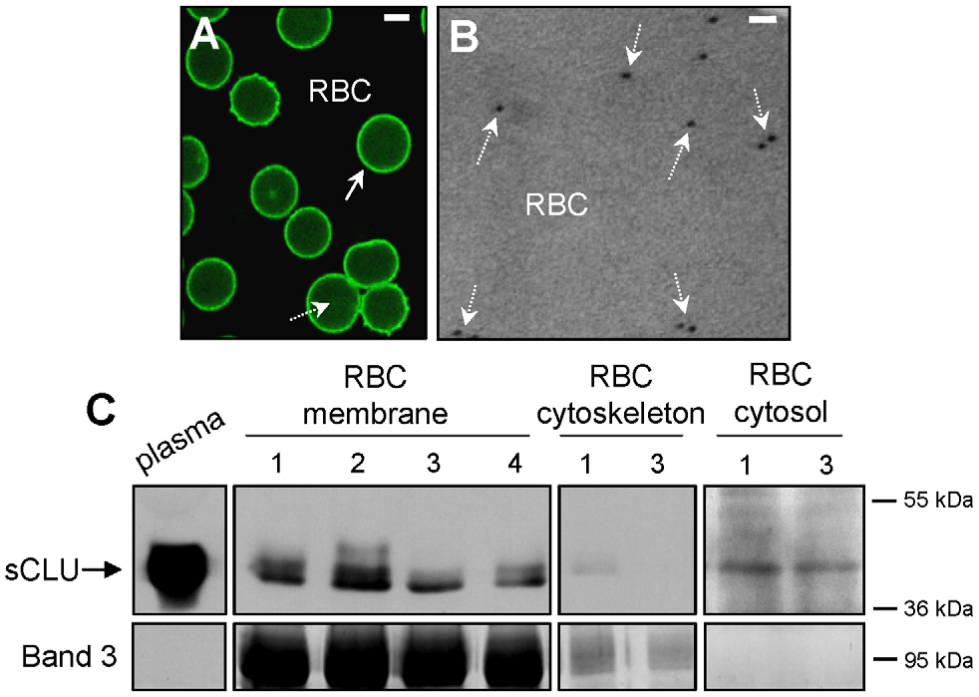
sCLU is a structural component of human RBCs plasma membrane and cytosol. (A) CLSM immunofluorescence or (B) TEM immunogold localization of sCLU in RBCs derived from healthy donors (representative of preparations in 3 or 2 subjects, respectively). Solid (A) or dashed (A and B) arrows indicate sCLU localization at the periphery of RBCs or intracellularly, respectively. Bars, CLSM, 3 µm; TEM, 100 nm. (C) Representative immunoblot analysis of isolated plasma (5 µg of total protein per lane), membrane (20 µg), cytoskeleton (20 µg) and cytosol (200 µg) purified RBCs fractions probed with an anti-sCLU antibody. Band 3 probing was used to demonstrate fraction purity and equal protein loading; numbers 1 to 4 denote different subjects (out of the 18 tested). Molecular weight markers are shown to the right of the blots.

Previous studies suggested that sCLU distributes in human RBCs [21]. Therefore, we investigated by means of light and electron microscopy as well as by biochemical cellular fractionation sCLU distribution in RBCs derived from healthy subjects. sCLU *in situ* localization analysis by both CLSM immunofluorescence (N = 3, Fig. 1A) and TEM immunogold (N = 2, Fig. 1B) showed that the majority of sCLU-specific labelling was located on the membrane of the RBCs (solid arrows; Fig. 1A); also a certain proportion of labelling occurred at the cytosol (dashed arrows; Figs 1A and B). To confirm the microscopical findings by biochemical analysis we fractionated the isolated RBCs into purified plasma membrane, cytoskeleton and cytosol preparations (N = 18, 21-42 years old, on average 31±8.3 years old, females/males ratio = 1). Immunoblotting analysis revealed that the majority of the mature sCLU heterodimer (reduced in two bands of ∼40 kDa) [16] co-isolated with the total membrane preparations while a small proportion of RBCs sCLU was also found at the cytosol (Fig. 1C), in all the subjects examined (N = 18). As cytoskeleton fractions were free of sCLU immunoreactivity (Fig. 1C), we concluded that sCLU is a major component of the non-cytoskeletal parts of the RBCs membrane and a minor component of RBCs cytosol.

### sCLU localizes at both the extra- and intracellular sides of the human RBCs membrane

**Figure 2:**
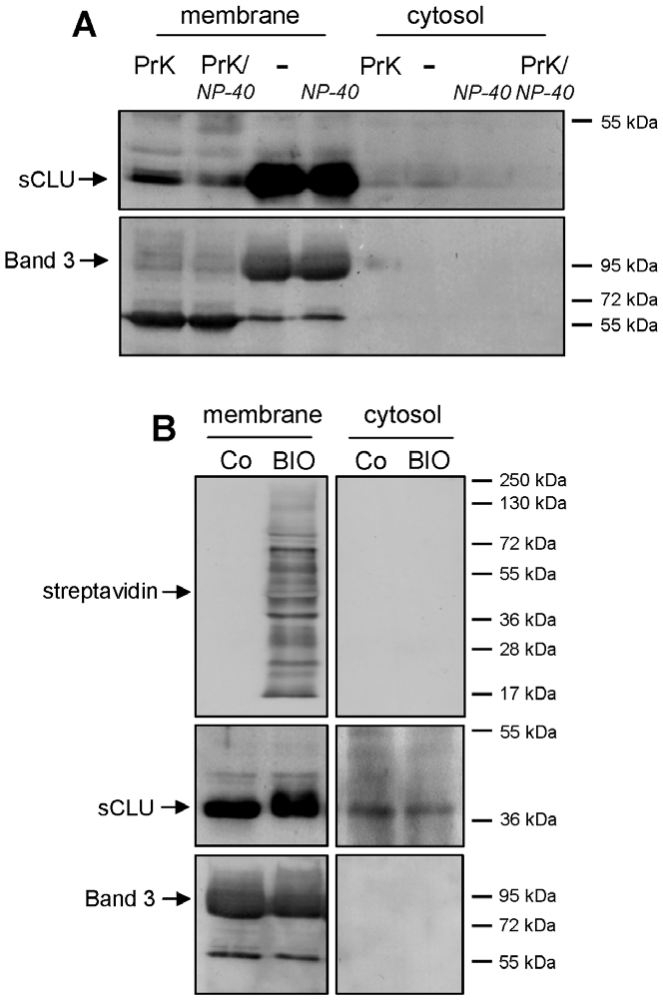
RBCs sCLU localizes at both extra- and intracellular sides of the plasma membrane. (A) Representative immunoblot analysis of purified membrane and cytosol fractions of untreated RBCs (-); RBCs treated with NP-40 (NP-40) or RBCs digested with proteinase K in the absence (PrK) or presence of NP-40 (PrK/NP-40). Samples (N = 3) were probed with specific anti-sCLU and anti-Band 3 antibodies. (B) Immunobloting of streptavidin, sCLU and Band 3 in purified membrane and cytosolic fractions of control (Co) or biotinylated (BIO) RBCs (N = 3). Molecular weight markers are shown to the right of the blots.

Τo further investigate the topology of sCLU we sought to answer the question of whether the membrane-associated sCLU localizes at the extracellular and/or the intracellular sides. Thus, we digested the extracellular proteins of intact RBCs with the highly active and unspecific proteinase K and we examined the sCLU content of purified membrane and cytosolic preparations (N = 3, 24.3±4.0 years old subjects, females/males ratio = 0.5). As shown in Fig. 2A, the membrane expression of sCLU in proteinase K-treated RBCs was significantly decreased compared to the non-treated RBCs. Interestingly, and in contrast to the transmembrane protein Band 3 which was totally reduced to a main (intracellularly protected) proteolytic fragment of 60 kDa, the sCLU plasma membrane immunoreactivity was not entirely eliminated following RBCs exposure to proteinase K (Fig. 2A). This finding indicated that the RBC membrane-associated sCLU localizes at both the extracellular (exposed to proteinase K) as well as the cytosolic (protected from proteinase K) sides of the RBC membrane. As expected, the amount of cytosolic sCLU remained unchanged after the extracellular digestion of intact RBCs verifying that cytosolic sCLU represents a membrane-independent component. In support, proteinase K treatment of RBCs in the presence of NP-40 (extra- and intracellular unspecific protein digestion) resulted in further reduction of the membrane-bound sCLU while the cytosolic molecules were entirely eliminated (Fig. 2A).

To exclude the possibility that the cytosolic sCLU originates from the membrane-associated protein molecules which redistribute from the cellular surface to the cytosol during the RBCs osmotic lysis (applied for the biochemical preparations of membrane and cytosol fractions) we labelled the surface of intact RBCs with biotin prior to their osmotic lysis (N = 3, the same group of subjects). As shown in the representative experiment of the Fig. 2B, the cytosolic preparations were completely free of biotinylated membrane proteins. Conclusively, sCLU is a novel structural component of both the extracellular and cytoskeleton-free cytosolic sides of the human RBCs plasma membrane.

### The RBCs membrane levels of sCLU decrease significantly during organismal aging or following organism exposure to exogenous stress

**Figure 3:**
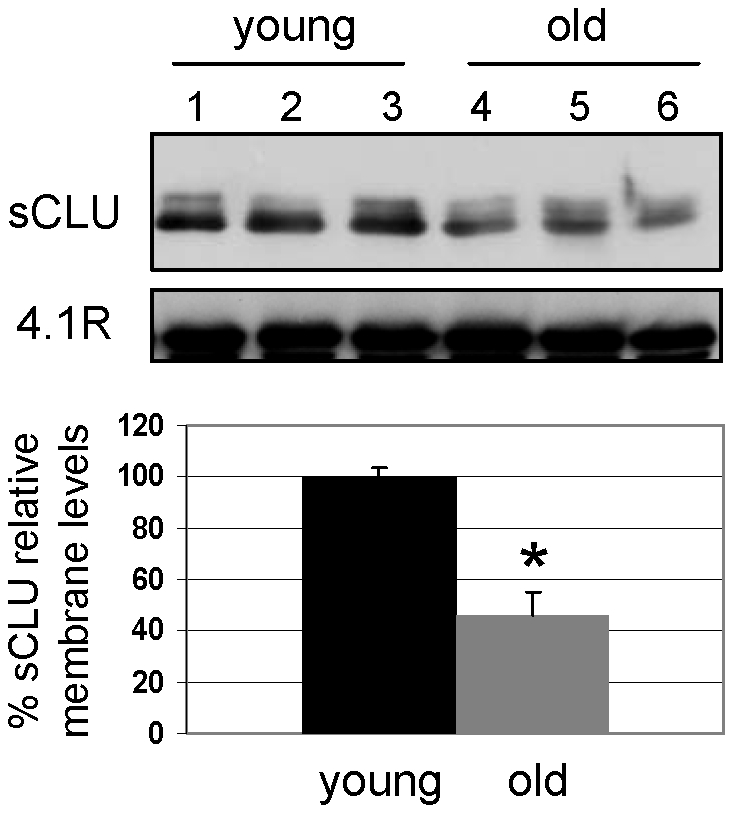
RBCs sCLU membrane levels are decreased during *in vivo* organismal aging. Representative immunoblot analysis and collective densitometry of sCLU relative levels at the membrane of RBCs derived from young (N = 13) or old (N = 10) non-smoking healthy subjects. Probing with anti-4.1R protein was used as reference for equal protein loading. Densitometric data were normalized against the sCLU values of the young donors. Error bars, ±s.d; asterisk, significance at p<0.05.

We then focused on the analysis of the sCLU membrane levels during *in vivo* organismal aging. For that purpose we studied purified RBCs from young (N = 13, 24.1±2.2 years old) and old (N = 10, 82±4.9 years old) healthy subjects. Our analysis revealed a more than 50% decrease in the sCLU membrane levels of RBCs derived from elderly people as compared to the young subjects (p<0.05, Fig. 3). This finding clearly indicated that during *in vivo* organismal aging, a condition of increased oxidative stress and accelerated or disturbed RBCs senescence [4], [29], the RBCs membrane sCLU content is reduced.

**Figure 4:**
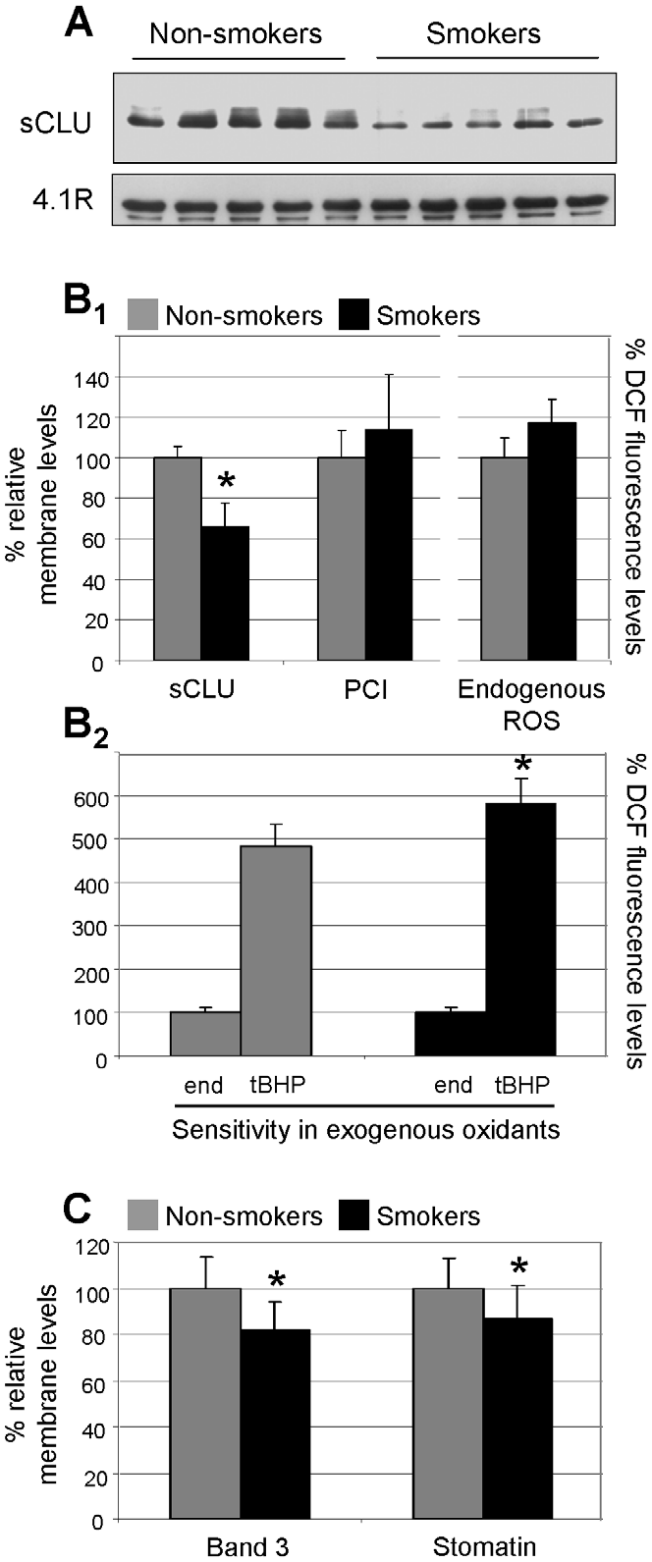
Decreased sCLU membrane levels in the RBCs of (otherwise healthy) smokers. (A) Representative immunoblot analysis of sCLU membrane levels in RBCs derived from healthy middle-aged cigarette smoking (N = 8) or non-smoking (N = 8) subjects (upper panel). 4.1R protein probing (lower panel) was used as reference for equal protein loading. (B_1_) Collective densitometric analysis of sCLU relative membrane content; PCI (data from not shown immunoblots) and endogenous cellular ROS from the smoking and non-smoking subjects. (B_2_) ROS measurement in RBCs from smokers and non-smokers challenged with exogenous tBHP (100 µM). (C) Densitometric analyses of Band 3 and stomatin relative membrane levels (data from not shown immunoblots). Presented data for sCLU, Band 3 and stomatin are mean values of each protein relative proportion against a reference membrane protein (4.1R) followed by normalization to samples derived from controls (non-smokers). ROS values represent the mean ± s.d. of dichlorofluorescein (DCF) fluorescence levels of two independent experiments (done in triplicates) following normalization to a standard protein quantity. Values represent the relative percentage to either the non-smokers (control) (Fig. B_1_) or to the endogenous ROS (Fig. B_2_) measurements. Error bars, ±s.d.; asterisks, significance at p<0.05.

Since prolonged intense smoking is thought to be a source of continuous, acute oxidative stress, we then investigated the membrane levels of sCLU in the RBCs of heavy middle-aged cigarette smokers. By using immunoblotting analysis, we showed that the membrane levels of sCLU in the RBCs derived from cigarette smokers (N = 8, 39.4±2.4 years old) were significantly reduced (by ∼34%, p<0.05) compared to age-matched non-smoking subjects (N = 8, 39.9±1.8 years old, Figs 4A, 4B_1_). To examine whether sCLU reduced levels in smokers' RBCs correlate with increased levels of oxidative stress we assayed the membrane proteome carbonylation index (PCI) as well as the ROS levels in RBCs from non-smoking and smoking subjects. Compared to the non-smokers' RBCs, the PCI and the ROS levels increased by ∼20% in the smokers' samples (Fig. 4B_1_). Moreover, as shown in Fig. 4B_2_ the RBCs of smoking donors were more susceptible to exogenous oxidants. Specifically, addition of tBHP increased their cellular oxidative load by almost 5.8-fold (induction in control RBCs was ∼4.8-fold on average). Thus, under *in vivo* conditions, the RBCs of the smoking donors are under sustained increased oxidative stress. Notably, a negative correlation between sCLU membrane levels and ROS accumulation was observed (2-tailed Spearman's rho correlation coefficient r = -0.800, p<0.010). Finally, by means of immunoblotting analysis the amount of two membrane proteins with unambiguous role in RBCs senescence and vesiculation, namely Band 3 and stomatin [2], was also found to be significantly reduced in the membrane of cigarette smokers (Fig. 4C); as for the sCLU, the RBCs membrane stomatin levels correlated negatively with the endogenous ROS levels (r = -0.802, p<0.010). These observations clearly suggested an accelerated aging and/or membrane vesiculation rate in the oxidized RBCs of the smoking donors.

### Decreased sCLU protein levels in the oxidized RBCs membrane of patients with hemolytic anemia

**Figure 5:**
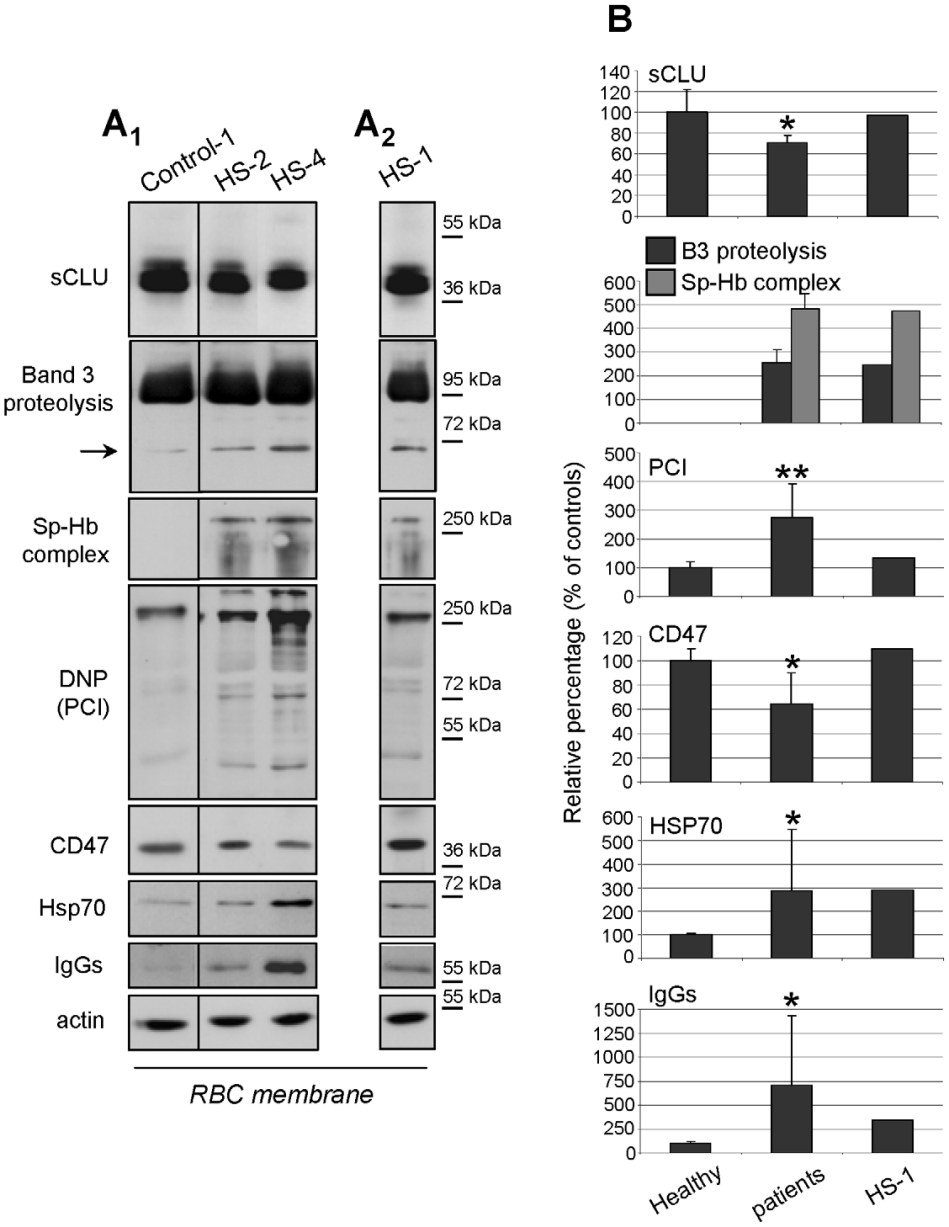
Reduced sCLU membrane levels in hemolytic anemia correlate to markers of RBCs senescence, redox imbalance and erythrophagocytosis. (A_1_) Representative immunoblots of RBCs plasma membrane preparations from a healthy subject (Control-1) and two patients with hereditary spherocytosis (HS-2 and HS-4) exhibiting decreased sCLU membrane levels. (A_2_) Representative immunoblot analysis of a RBCs membrane preparation from the splenectomized patient HS-1; sCLU membrane levels are similar to controls. Immunoblots were also probed with antibodies against Band 3, Hb, dinitrophenylhydrazone (DNP) residues (oxyblot analysis), CD47, Hsp70, IgGs and actin (used as loading control); in some cases compositions of different blots is shown. Molecular weight markers are indicated at the right of each blot. (B) Densitometric analyses of sCLU and cellular senescence or oxidative stress markers in respective immunoblots. Shown data are the mean values of the proteins' relative proportion against a reference membrane protein followed by normalization to the controls (N = 10, non-smoking, age-matched subjects). Error bars, ±s.d.; single or double asterisks, difference of patients vs. controls at significance level of p<0.05 or p<0.01, respectively.

The delineated functional role of sCLU in RBCs senescence was further investigated at conditions of *in vivo* endogenously stressed RBCs. Therefore, we obtained RBCs from patients with congenital hemolytic anemia; a pathological condition being characterized by accelerated senescence, severe oxidative stress [30] and increased vesiculation rate of erythrocytes [2], [5]. As shown in Fig 5 all patients examined [except for HS-1 (splenectomized)] exhibited decreased levels of the RBCs-membrane-associated sCLU (relative percentage range 63-79%, N = 9, p = 0.032 vs. controls) compared to an equal number of age-matched healthy controls. sCLU membrane levels in the case of splenectomised patient HS-1 (offspring of HS-4, Fig. 5A_2_ and Fig. 5B) were close to normal (∼97.1% of the controls). We then examined the correlation of sCLU levels with a number of well-established RBCs aging membrane markers. As it is clear from Figure 5, the aging-related modifications of Band 3 and spectrin, namely Band 3 partial proteolysis and spectrin/Hb complex formation [7], [9], [29] were exclusively detected in the patients and not in healthy controls (Figs 5A, 5B). Immunoblot analysis (Fig. 5) revealed that all patients (except for HS-1) exhibited pathologically increased (p<0.010 vs. controls) membrane PCI. The observed carbonylation levels were inversely correlated to the sCLU membrane levels (N = 10, 2-tailed Spearman's rho correlation coefficient r = -0.699, p = 0.024). Moreover, sCLU variation correlated positively with that of the antigenic marker CD47 (N = 10, 2-tailed Spearman's rho correlation coefficient r = 0.736, p = 0.015) and negatively with the significantly increased IgGs (N = 10, p<0.05 vs. controls) as well as the Hsp70 and Band 3 proteolytic fragments; those correlations were not statistically significant (p<0.08), probably due to the small number of the patients examined. In conclusion, decreased *in vivo* membrane levels of sCLU in RBCs of patients with congenital hemolytic anemia correlated with membrane proteome carbonylation, erythrophagocytosis marks and RBCs aging-related modifications.

### *In vivo* senescence of RBCs results in significant loss of sCLU from plasma membrane

**Figure 6:**
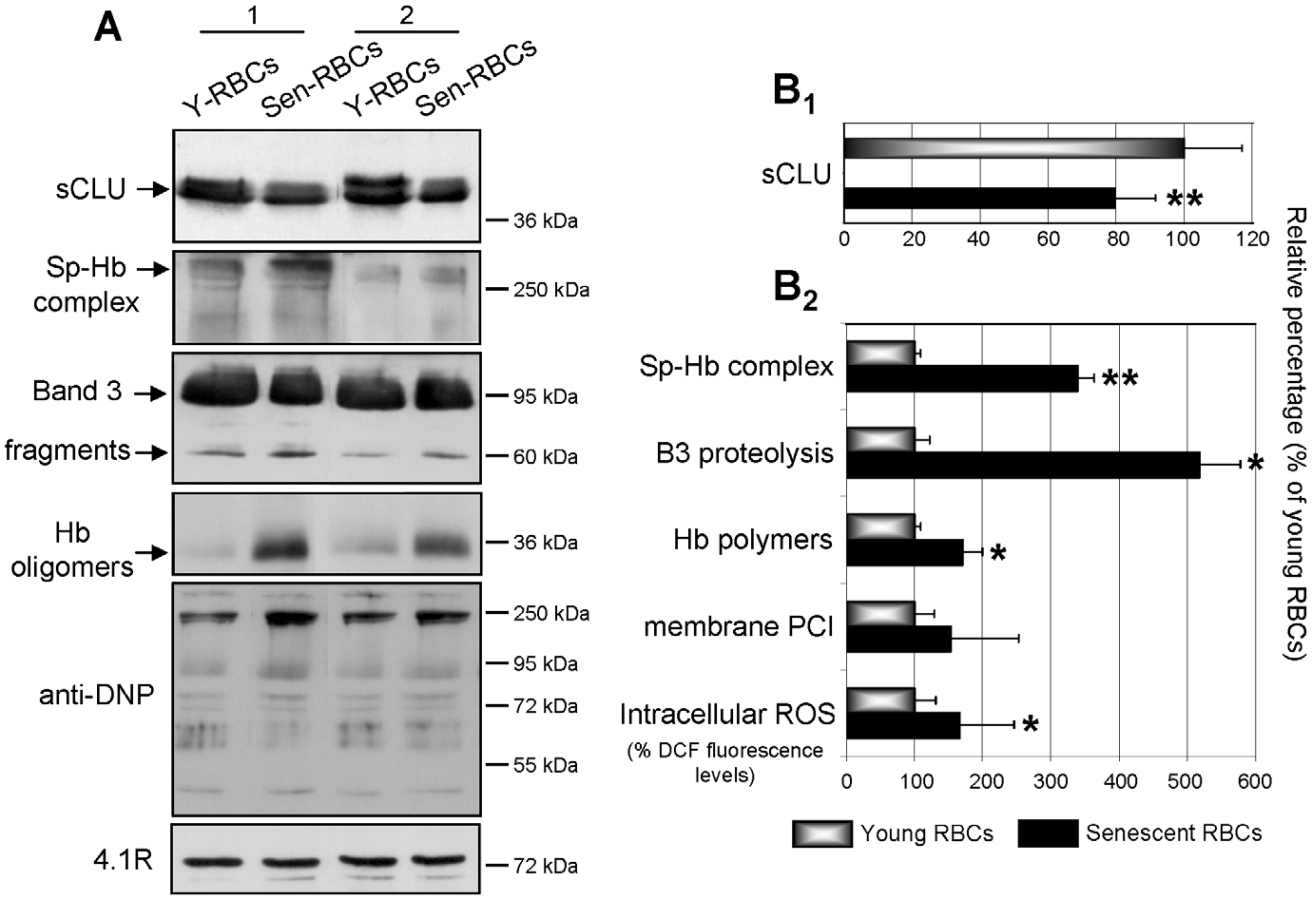
Loss of membrane sCLU during *in vivo* RBCs senescence. Analysis of sCLU membrane content, as well as of cellular aging and oxidative stress markers in young and senescent RBCs. (A) Representative immunoblots of membrane preparations from young (Y) and senescent (Sen) RBCs fractionated from the peripheral blood of two subjects (1 and 2); blots were probed with antibodies against sCLU, spectrin, Band 3, Hb, dinitrophenylhydrazone (DNP) moiety and 4.1R protein (used as loading reference). Molecular weight markers are shown to the right of the blots. (B) Densitometric analyses of sCLU (B1) and aging or oxidative stress markers presentation (B2) in respective immunoblots from middle-aged non-smoking volunteers (N = 6). Presented data are mean values of each protein relative proportion against a reference membrane protein (4.1R) followed by normalization to the young RBCs (set to 100% of RBCs membrane sCLU content). ROS values represent the mean ± s.d. of dichlorofluorescein (DCF) fluorescence levels of six independent experiments (done in triplicates) following normalization to a standard protein quantity. Error bars, ±s.d.; single or double asterisks indicate difference of young vs. senescent RBCs at p<0.05 or p<0.01, respectively.

As we had previously established a critical role for sCLU in the cellular senescence of many human cell types [16] we investigated a probable similar implication in the cellular senescence of mature RBCs. Thus, we studied the membrane levels of sCLU in young and senescent RBCs. Cell preparations [N = 6, middle-aged (39.1±4.3 years old), non-smoking subjects] were obtained by fractionation of RBCs according to their density; this is a physical method that yields cellular fractions enriched with old (higher density) or younger (lower density) RBCs. The fractionation of the erythrocyte suspensions was demonstrated by density-related changes in the erythrocyte indexes MCV and MCHC (MCV 96.5±12.5fL to 84.1±5.4fL and MCHC 29.6±1.6 g/dL to 34.5±2.4 g/dL for young and senescent RBCs, respectively). To further validate the fractionation, the derived young and senescent preparations were examined for the presence of well-established proteome aging markers in the RBCs membrane, such as the formation of spectrin-Hb complexes and the proteolysis of Band 3 (Fig. 6A and 6B_2_). Indeed, compared to the young cells, the senescent RBCs exhibited increased expression of both aging-related markers (Fig. 6) (p = 0.009 for the spectrin-Hb complexes and p = 0.049 for the Band 3 proteolysis). All of the senescent RBCs preparations examined were characterized by an average decrease of ∼20±2.2% in their membrane sCLU content (p = 0.004 compared to young RBCs) (Figs 6A, 6B_1_). Previous studies have suggested that erythrocyte senescence is accompanied by a decrease in the antioxidant response [31]. In support, we detected an increase in the intracellular ROS levels (165±24% in senescent vs. 100±8 in young cells; p = 0.031) during *in vivo* RBCs senescence. Moreover, we found a substantial increase in both the membrane-bound oxidized/denatured Hb species (171±28% in senescent vs. 100±9 in young cells) and PCI (152±60% in senescent vs. 100±22 in young cells) (Fig. 6); only the former variation was significant (p = 0.014). Conclusively, RBCs *in vivo* senescence is accompanied with significant losses in the membrane sCLU content. This decrease parallels an enhanced expression of aging and oxidation markers.

## Discussion

Despite the fact that sCLU [15] as well as apolipoprotein E [32] have been previously implicated in erythroid differentiation, the role of apolipoproteins in erythrocytes physiology, maturation and senescence remains largely unknown. Our results corroborated previous preliminary studies suggesting that sCLU is a component of mature human RBCs [21], [33]. Following our thorough examination we report that sCLU is a structural component of both the extra- and (non-cytoskeletal) intracellular sides of RBCs membrane and it also localizes at the cytosol. We and others have previously reported that although sCLU is primarily considered a secreted protein it might also act intracellularly having a vital role in the maintenance of cellular homeostasis and proteome stability of human cells [21], [34]-[36]. Thus, sCLU may represent the only known chaperone exerting both an extra- and intracellular function. Our reported findings in RBCs provide additional evidence for sCLU localization and probable function in cytosolic cellular compartments. Considering that human erythrocytes are incapable of protein synthesis, intracellular membrane and cytosolic sCLU is most probably inherited from precursor erythroid cells. On the other hand, the sCLU molecules that bind to extracellular sides of RBCs membrane may originate either from the plasma circulating sCLU that binds to the RBCs IgGs or from endogenous sources during RBCs maturation. The increased binding of IgGs in the RBCs of patients with hemolytic anemia showing decreased sCLU membrane levels suggests that the extracellular membrane association of sCLU in RBCs may depend on factors other than IgG binding.

An important question regarding erythrocytic sCLU referred to whether it has any functional role in those highly specialized cells or it simply represents a non-functional vestige from erythroid precursor cells. As previous reports in typical nucleated cells showed sCLU functional involvement in oxidative-stress responses and cellular senescence [16], we asked whether sCLU is actively involved in similar processes in mature RBCs. Our findings suggest that sCLU in human RBCs is a sensitive biosensor of both increased oxidative stress and cellular senescence. Specifically, the significant losses of sCLU seen in stressed or senescent RBCs paralleled that of prominent cellular senescence markers [7], [9], like Band 3 fragmentation and formation of the spectrin-Hb complexes. Moreover, in hemolytic cases, the membrane levels of sCLU were positively correlated with those of CD47 protein that is reduced in cases of hereditary anemias [37] and during *in vitro* aging of human RBCs [33]. The inverse correlation of sCLU levels with those of the pathologically increased RBCs membrane-bound IgGs further suggests an association of sCLU presentation with the phagocytosis rate of diseased RBCs [5], [7].

The fact that RBCs membrane remodelling during organismal aging and cellular senescence exhibit similar molecular characteristics [2], [5], [38] along with the identical regulation of sCLU in RBCs during organismal aging or in cellular senescence, indicates an active role of sCLU in senescing RBCs. RBCs senescence, as in many other cell types, is mainly driven by oxidative effects [31], [39]. The significant decrease of sCLU at the membrane of both healthy and defected RBCs was consistently associated with increased RBCs susceptibility to exogenous oxidants and with measurable manifestations of redox imbalance, namely, increased intracellular ROS accumulation, membrane proteome carbonylation and binding of oxidized/denatured Hb species to the membrane. These observations were further accentuated in the cases of healthy smokers or patients with hemolytic anemia, where the membrane levels of sCLU were negatively correlated with the proteome carbonylation index (PCI) and the intracellular ROS levels, respectively.

Cigarette smoking has been associated with increased oxidation of RBCs and plasma although it seems that, at least, in young smokers it also activates the RBCs antioxidant mechanisms as an adaptation process to counteract the oxidant factors [40], [41]. Our findings by documenting a relatively small increase (∼20%) in the PCI and ROS accumulation in RBCs from middle aged smokers corroborate these previous reports. Nevertheless, even this relatively small increase imposes a sustained cellular stress as indicated by the increased RBCs susceptibility to exogenous oxidants. Therefore, one can postulate an accelerated rate of RBCs vesiculation in these samples as a protective mechanism against exogenous oxidative stress. In support to this assumption, RBCs membranes from smokers do not retain normal Band 3 and stomatin expression levels. The positive correlation of vesicle-associated stomatin with sCLU suggests that both proteins are commonly affected by the intracellular oxidative stress imposed by the cigarette smoke. Considering, the fact that during organismal aging or at several diseases that represent states of increased oxidative stress (e.g. diabetes type II, atherosclerosis or Alzheimer's disease) the sCLU concentration in human plasma is elevated [42], [43] we conclude that the reduced sCLU membrane levels found in RBCs from smokers or patients with hemolytic anemia (see below) are related to endogenous RBCs-specific molecular processes.

Erythrocytes senescence and the consequent accumulation of ROS [31] cause oxidative modifications on proteins which are accompanied by a loss of protein function [29], [44]. These modified or damaged proteins (often termed as “client proteins”) are in need of assistance by chaperones and are mostly exocytosed to RBCs-derived vesicles which have been found to contain a wide range of damaged or potentially damaging molecules [7]. The protective role of molecular chaperones in these processes has been previously exemplified for Hsp70 in stressed RBCs where Hsp70 was found to stabilize the partially defected cytoskeletal proteins [45]. RBCs vesiculation constitutes a mechanism for the removal of erythrocyte membrane patches containing oxidative lesions or death signalling molecules, thereby postponing the untimely elimination of otherwise healthy erythrocytes [46]. This process is an integral component of the normal cellular senescence and it is accelerated during *in vivo* aging, organism exposure to acute oxidative stress or in diseases like congenital hemolytic anemia [2], [7], [47]. We propose that the sCLU removal from the RBCs membrane during senescence or RBCs exposure to increased oxidative stress takes place via the process of vesiculation. Considering that sCLU functions as a chaperone involved in the quality control of protein folding [13] and in the clearance of cellular debris by non-professional phagocytes [48] we assume that it also contributes to the scavenging of oxidized or aggregated molecules that are selectively removed from senescent or stressed RBCs via vesiculation. Additional confirmatory evidence comes from the splenectomized patient HS-1. This patient exhibits normal sCLU membrane levels while his father (patient HS-4), who carries the same genetic defect, is severely deficient in sCLU. The emerging beneficial influence of splenectomy in the maintenance of normal membrane sCLU levels is probably related to the role of spleen in facilitating the aging-associated vesicle formation [2]. Indeed, in the absence of a functional spleen, the normal increase of vesiculation in the second half of the erythrocyte lifespan does not occur [2]. Apart from the vesiculation, splenectomy further ameliorates the severity of anemia and the clinical severity of hereditary spherocytosis by increasing the circulatory life span of spherocytes, especially in the cases of spectrin- or ankyrin-deficient patients [47]. Therefore, the almost normal levels of membrane-associated sCLU in the splenectomised patient HS-1 most probably relates to a decreased release of sCLU-containing vesicles.

Taken together, our results provide novel evidence for an emerging role of sCLU, a novel structural component of RBCs plasma membrane and cytosol, in the physiology of mature human erythrocytes. The erythrocytes sCLU content decreases significantly *in vivo* in response to cellular senescence and oxidative stress. Although the primary underlying stimulus and mechanism that drives or mediate respectively, the decrease in sCLU levels remain elusive, the currently reported data support the characterization of the sCLU as a sensitive molecular biomarker of senescence and oxidative stress in erythrocytes. Our findings are of considerable importance especially under the light of the recently increased focus on the signalling molecules and mechanisms operating in mature RBCs during senescence or following exposure to endogenous/exogenous stress stimuli.
